# Supplementary material for: G1-Cyclin2 (Cln2) promotes chromosome hypercondensation in eco1/ctf7 rad61 null cells during hyperthermic stress in Saccharomyces cerevisiae
Source: G3 (Bethesda). 2022 Jun 23;12(8):jkac157. doi: 10.1093/g3journal/jkac157 (PMC9339302; doi:10.1093/g3journal/jkac157)
Supplement: jkac157_Supplemental_Table_1 [file jkac157_supplemental_table_1.docx]

**Supplemental Table 1:** List of yeast strains used in this study.

Multiple isolates, independently derived through transformation, are indicated along with their placement in figures.

**YPH499** (Sikorski and Hieter, 1989)

*MATa ura3-52 lys2-801_amber ade2-101_ochre trp1-Δ63 his3-Δ200 leu2-Δ1*

**YBS255** (Skibbens et al., 1999)

*MATa CTF7:LEU2 ctf7::HIS3 ade2-101 his3∆200 leu2∆1 lys2-801 trp1∆63 ura3-53*

**YBS514** (Skibbens et al., 1999)

*MATa ctf7-203 ctf7::HIS3 ade2-101 his3∆200 leu2∆1 lys2-801 trp1∆63 ura3-53*

**YMM808** (Maradeo and Skibbens, 2010)

*MATa rad61::URA3 ade2-101 his3Δ200 leu2Δ1 lys2-801 trp1Δ63 ura3-52*

**YMM828** (Maradeo and Skibbens, 2010)

*MATalpha cft7::HIS3 rad61::URA3 ade2-101 his3∆200 leu2∆1 lys2-801 trp1∆63 ura3-52*

**YMM829** (Maradeo and Skibbens, 2010)

*MATa cft7::HIS3 rad61::URA3 ade2-101 his3∆200 leu2∆1 lys2-801 trp1∆63 ura3-52*

**YBS3543** (isolate V) (Current study, Fig.1B)

*MATa cft7::HIS3 rad61::URA3 cln2::KAN ade2-101 his3∆200 leu2∆1 lys2-801 trp1∆63 ura3-52*

**YBS3544**  (Current study, Fig.1B)

*MATa cft7::HIS3 rad61::URA3 cln2::KAN ade2-101 his3∆200 leu2∆1 lys2-801 trp1∆63 ura3-52* (isolate VI)

**YBS3545**  (Current study, Fig.1B)

*MATa cft7::HIS3 rad61::URA3 CLN2 ade2-101 his3∆200 leu2∆1 lys2-801 trp1∆63 ura3-52 KAN* (isolate VII)

**YBS3550**  (Current study, Fig.1C)

*MATa cft7::HIS3 rad61::URA3 cln2::KAN ade2-101 his3∆200 leu2∆1 lys2-801 trp1∆63 ura3-52* (isolate 1)

**YBS3552**  (Current study, Fig.1C)

*MATa cft7::HIS3 rad61::URA3 cln2::KAN ade2-101 his3∆200 leu2∆1 lys2-801 trp1∆63 ura3-52* (isolate 2)

**YBS3553** (Current study, Fig.1C)

*MATa cft7::HIS3 rad61::URA3 cln2^1-221^::KAN ade2-101 his3∆200 leu2∆1 lys2-801 trp1∆63 ura3-52* (isolate 1)

**YBS3554** (Current study, Fig.1C)

*MATa cft7::HIS3 rad61::URA3 cln2^1-221^::KAN ade2-101 his3∆200 leu2∆1 lys2-801 trp1∆63 ura3-52* (isolate 2)

**YBS3555** (Current study, Fig.3)

*MATa ctf7-203 ctf7::HIS3 cln2::KAN ade2-101 his3∆200 leu2∆1 lys2-801 trp1∆63 ura3-53* (isolate 1)

**YBS3556** (Current study, Fig.3)

*MATa ctf7-203 ctf7::HIS3 cln2::KAN ade2-101 his3∆200 leu2∆1 lys2-801 trp1∆63 ura3-53* (isolate 2)

**YBS3557** (Current study, Fig.3)

*MATa ctf7-203 ctf7::HIS3 cln2::KAN ade2-101 his3∆200 leu2∆1 lys2-801 trp1∆63 ura3-53* (isolate 3)

**YBS3570** (Current study, Fig.2B)

*MATa cft7::HIS3 rad61::URA3 cln1::KAN ade2-101 his3∆200 leu2∆1 lys2-801 trp1∆63 ura3-52* (isolate 1)

**YBS3571** (Current study, Fig.2B)

*MATa cft7::HIS3 rad61::URA3 cln1::KAN ade2-101 his3∆200 leu2∆1 lys2-801 trp1∆63 ura3-52* (isolate 2)

**YBS3572** (Current study, Fig.2B)

*MATa cft7::HIS3 rad61::URA3 cln1::KAN ade2-101 his3∆200 leu2∆1 lys2-801 trp1∆63 ura3-52* (isolate 3)

**YBS3576** (Current study, Fig.2A)

*MATa cft7::HIS3 rad61::URA3 cln3::KAN ade2-101 his3∆200 leu2∆1 lys2-801 trp1∆63 ura3-52* (isolate 1)

**YBS3577** (Current study, Fig.2A)

*MATa cft7::HIS3 rad61::URA3 cln3::KAN ade2-101 his3∆200 leu2∆1 lys2-801 trp1∆63 ura3-52* (isolate 2)

**YBS3578** (Current study, Fig.2A)

*MATa cft7::HIS3 rad61::URA3 cln3::KAN ade2-101 his3∆200 leu2∆1 lys2-801 trp1∆63 ura3-52* (isolate 3)

**YBS3611** (Current study, Fig.4)

*MATa cft7::HIS3 rad61::URA3 ade2-101 his3∆200 leu2∆1 lys2-801 trp1∆63 ura3-52 pRS315* (isolate 1)

**YBS3612** (Current study, Fig.4)

*MATa cft7::HIS3 rad61::URA3 ade2-101 his3∆200 leu2∆1 lys2-801 trp1∆63 ura3-52 pRS315* (isolate 2)

**YBS3613** (Current study, Fig.4)

*MATa cft7::HIS3 rad61::URA3 ade2-101 his3∆200 leu2∆1 lys2-801 trp1∆63 ura3-52 pBS103(POL30^OE^,* Skibbens et al., 1999*)* (isolate 1)

**YBS3614** (Current study, Fig.4)

*MATa cft7::HIS3 rad61::URA3 ade2-101 his3∆200 leu2∆1 lys2-801 trp1∆63 ura3-52 pBS103(POL30^OE^,* Skibbens et al., 1999*)* (isolate 2)

**YBS3615** (Current study, Fig.4)

*MATa cft7::HIS3 rad61::URA3 ade2-101 his3∆200 leu2∆1 lys2-801 trp1∆63 ura3-52 pBS102(POL30^OE^,* Skibbens et al., 1999*)* (isolate 3)
