# Supplementary material for: G1-Cyclin2 (Cln2) promotes chromosome hypercondensation in eco1/ctf7 rad61 null cells during hyperthermic stress in Saccharomyces cerevisiae
Source: G3 (Bethesda). 2022 Jun 23;12(8):jkac157. doi: 10.1093/g3journal/jkac157 (PMC9339302; doi:10.1093/g3journal/jkac157)
Supplement: jkac157_Supplemental_Table_2 [file jkac157_supplemental_table_2.docx]

**Supplemental Table 2:** List of primers used in this study.

**oRVS23** internal *KANMX*

5' GAT TGT CGC ACC TGA TTG CC 3'

**oRVS3194** Forward - *CLN2/Longtine vector*

5' CCA CAT CAT AAT TTG CAT ACA AAA GAA AAT CTT TTC TTT TTT CCA TTC CGG ATC CCC GGG TTA ATT A 3'

**oRVS3195** Forward - *CLN2 truncation/Longtine vector*

5' GAT GAA AAT TGT TTG ATG CAA TAC GAA CTT TAT GAA ATT CAA GTT CGG ATC CCC GGG TTA ATT A 3'

**oRVS3196** Reverse - *CLN2/Longtine vector*

5' GGT ACG TTT GGC AAA TTG GCA TTC ATT TAT CAT GAA AAG AAC AGG AAG AAT TCG AGC TCG TTT AAA C 3'

**oRVS3197** Forward - *CLN2*

5' CCT CTT ACT GGT TTT TTA GTG 3'

**oRVS3198** Forward - *CLN1*

5' GAA TTG GTA ATG CTT ATC TTC 3'

**oRVS3199** Forward - *CLN1/Longtine vector*

5' ACG ATA ACA ATA ATA GCA ATT AAA TAA AAT AGC ACT ACC ACC ACT CCA CTG CTC CGG ATC CCC GGG TTA ATT TAA 3'

**oRVS3200** Reverse - *CLN1/Longtine vector*

5' AGC GTA GTA TTC CGT TAT TAA TTA AGT ATA TAT GTA GGC TTG ATG AGA AAA TGG GAA TTC GAG CTC GTT TAA AC 3'

**oRVS3201** Forward - *CLN3*

5' CCT TTT TCT ATT TCT CTT TTC 3'

**oRVS3202** Forward - *CLN3/Longtine vector*

5' GCA TTT CTT ACA TTC CAT TGC ATC TCC CTT TTA CTC TCG TTC AAG ACA CTG ATT CGG ATC CCC GGG TTA ATT AA 3'

**oRVS3203** Reverse - *CLN3/Longtine vector*

5' GTG AAA ACA AAA AGA AAA AAA GAT CAT TAA TGT ATG TTA ACG TAT TTG CTT TGC GAA TTC GAG CTC GTT TAA AC 3'
